# The Association Between Patient Engagement and Quality of Care Interactions Among Acute Care Patients With Dementia

**DOI:** 10.1093/geroni/igaf122.725

**Published:** 2025-12-31

**Authors:** Rachel McPherson

**Affiliations:** University of Maryland School of Nursing, Baltimore, Maryland, United States

## Abstract

Effective staff-patient interaction is critical to promote effective care delivery, which includes function-focused care to hospitalized persons with dementia. The purpose of this study was to determine whether the quality of staff-patient care interactions were associated with active patient engagement during the interaction after controlling for relevant covariates. The study was a secondary data analysis using baseline data from the Function Focused Care for Acute Care intervention study, with a total sample of 286 patients. Generalized linear mixed model indicated that there was a significant relationship between the quality of care interactions and patient engagement such that receiving positive care interactions resulted in higher odds of active patient engagement. These findings can inform future interventions and training for acute care staff to improve quality of care interactions and patient engagement.

